# Spine deformity board and the need for a multidisciplinary discussion of complex spine surgery cases: A proposal from the EANS young neurosurgeons committee

**DOI:** 10.1016/j.bas.2024.104145

**Published:** 2024-11-20

**Authors:** Luca Ricciardi, Stefan Motov, Gabriele Capo, Lorenzo Bertulli, Felix C. Stengel, Belo Diogo, Thomas Schoefl, Torstein R. Meling, Florian Ringel, Andreas K. Demetriades, Giovanni Raffa, EANS Young Neurosurgeons Committee

**Affiliations:** Neurosurgery Unit, Department of NESMOS, Sapienza University of Rome, Italy; Department of Neurosurgergy, Cantonal Hospital St.Gallen, St.Gallen, Switzerland; Neurochirurgia I, IRCCS Humanitas Research Hospital, Milan, Italy; Department of Neurosurgergy, Cantonal Hospital St.Gallen, St.Gallen, Switzerland; Servico de Neurochirurgia, Hospital Santa Maria, Unidade Local de Saude Santa Maria (ULSSM), Portugal; Clinic for Orthopaedic Surgery and Traumatology of the Musculoskeletal System, Cantonal Hospital St.Gallen, St.Gallen, Switzerland; Neurosurgery, Copenhagen University Hospital, Rigshospitalet, Denmark; Neurosurgery, University Medical Center (UMC), Mainz, Germany; Neurosurgery, NHS Lothian, Edinburgh University Hospitals, Edinburgh, Scottland, UK; Neurosurgery Unit, Department of BIOMORF, University of Messina, Italy

**Keywords:** Spine deformity, Scoliosis, Decisional algorithm, Multidisciplinary board, Surgical advancement

To the Editor,

This year the 13th Annual EANS Young Neurosurgeons Committee (YNC) meeting took place in Hamburg, Germany and was mainly focused on Spinal Oncology and Minimally Invasive Spine Surgery. It was an excellent opportunity for young neurosurgeons to share updates on current treatment concepts and exchange individual practices among different European neurosurgical departments, eventually providing a high level of panel discussions on essential spine topics under the guidance of senior experts from the EANS Spine Section.

This spine disorders meeting was one of the most attended and participated in the last years, providing novel ideas on managing challenging clinical scenarios such as adult spinal deformities (ASD) correction. Throughout the discussions during the lectures and case presentations, it has become evident that ASD needs a multidisciplinary decision-making process, including different medical specialties, to tailor the treatment to the individual patient characteristics. There was a general agreement on the usefulness of involving not only neurosurgeons and orthopedics but also pain therapists, radiologists, internists, neurophysiologists, psychologists, and physiotherapists in establishing a treatment plan. The proper surgical planning for ASD correction might require a thorough patient analysis, aiming to consider all clinical variables, e.g., psychological assessment, medical preconditions, pain pathway, etc., which should be included in the procedural workflow. Multidisciplinary board recommendations might guide the physician in charge of the patient to find the most suitable treatment and tailored approach for a complex condition like ASD (Friedman et al., 2020; Sethi et al., 2017).

The decision for scheduling an ASD patient for surgery should be based on both radiological criteria and the failure of standardized conservative treatments proposed and pursued for at least 3-to-6 months (Kreiner et al.; Atalay et al., 2023). Nonetheless, the spine surgeon usually manages these pre-surgical steps, aiming to evaluate the patient's condition individually and deciding single-handedly whether and when surgery is indicated. Whereas neurosurgeons might underestimate spine biomechanics and the global musculoskeletal impact, such as hips and knee mobility and global alignment, orthopedics tend to overstate the need for standard open instrumentation surgeries with long segment fixation, even in patients who may potentially benefit from less invasive approaches(McDonald et al., 2023).

On-site polling of the existing Spine Deformity Boards throughout the departments represented at the EANS YNC meeting demonstrated that only a few centers systematically discuss their cases in a multidisciplinary fashion. This finding confirms the general perception that, at the moment, in most centers, the single surgeon's experience alone determines the decision progress for ASD management. Accordingly, the lack of standardized criteria for a multidisciplinary discussion of these cases determines the absence of a decisional algorithm for ASD patients.

Nevertheless, a multidisciplinary spine board may help develop and standardize a decision-making process for various spine disorders. This might lead to more effective treatment protocols with better patient outcomes, lower complication rates, higher homogeneity in data collection and scientific reports interpretation, and lower risks for medico-legal issues. The proposal for a Minimally Invasive Spinal Deformity Surgery Algorithm (MISDEF) was initially developed and published by the International Spine Study Group in 2014 and then updated in 2019 (Mummaneni et al., 2014, 2019). These two algorithms suggest that surgical techniques might be applied according to deformity type and severity; however, neither data on conservative management protocols and their duration nor selection protocols for non-surgical cases have been included. Recently, alternative approaches have been introduced, such as short fixations limited to the lumbar spine or hybrid constructs using dynamic instrumentation, such as thoracic tethering above the lumbar fusion. Even the focal treatment of a limited number of unstable levels using only anterior and lateral approaches is now considered a legitimate option, further expanding the spectrum of surgical solutions (Agarwal et al., 2023). Accordingly, many procedures lack sufficient data to establish clinical or surgical superiority. Therefore, the only logical solution is to have dedicated boards for the multidisciplinary discussion of these cases.

Although guidelines on traumatic and degenerative spine disorders are currently available, the patient-specific parameters may require deviations from the standard practice. The surgical impact, especially in the adult and frail population, might have disastrous consequences depending on the strategy and execution. Furthermore, the comprehension of bone metabolic status, the musculoskeletal system, the responsiveness to medical protocols, the pre-and postoperative rehabilitation, and the psychological assessment of these patients are vital variables often underestimated.

In conclusion, we highlight the importance of conceptualizing institutional Spine Boards based on pathology in dedicated spine centers with a significant volume of spine procedures. We propose adopting standard protocols, eventually leading to a homogeneous case discussion, data collection, and consistency for scientific and clinical purposes. The involvement of AI and ML technologies could foster clinical-effective protocols in these patients. On the other hand, AI software has to be carefully considered, and expert human supervision and validation of these processes are strictly needed. Therefore, collecting and analyzing a consistent dataset from European institutions in the EANS framework should be pursued soon.

## Declaration of competing interest

The authors declare that they have no known competing financial interests or personal relationships that could have appeared to influence the work reported in this paper.
